# Complete genome sequence of *Priestia megaterium* CIMT4 strain isolated from maize rhizosphere

**DOI:** 10.1128/mra.01124-24

**Published:** 2025-06-20

**Authors:** Yohannes Ebabuye Andargie, Tino Flory Bashizi, Kyeongmo Lim, Eskindir Getachew, Jae-Ho Shin

**Affiliations:** 1 Department of Applied Biosciences, Kyungpook National University 34986, Daegu, Daegu, Republic of Korea; 2 Department of Plant Sciences, Bahir Dar University 128158, Bahir Dar, Amhara, Ethiopia; 3 NGS core facility, Kyungpook National University 34986, Daegu, Daegu, Republic of Korea; 4 Department of Integrative Biology, Kyungpook National University 34986, Daegu, Daegu, Republic of Korea; University of Strathclyde, Glasgow, United Kingdom

**Keywords:** rhizosphere, PGPR, *Priestia megaterium*

## Abstract

We sequenced the complete genome of *Priestia megaterium* CIMT4 isolated from the rhizosphere soil of maize (*Zea mays*) for its plant growth-promoting rhizobacteria (PGPR) characteristics. The strain has a genome size of 5.7-Mb, and the GC content is 37.9 %.

## ANNOUNCEMENT

*Priestia megaterium* (formerly *Bacillus megaterium*) is a gram-positive, rod-shaped bacterium reclassified under the genus Priestia (1). It functions as a biocontrol agent (2, 3) and plant growth promoter (4, 5), reducing reliance on synthetic fertilizers and pesticides. In addition, it contributes to environmental sustainability through polyhydroxybutyrate (PHB) production (6, 7) and detoxification of environmental and food contaminants (8, 9).

We isolated *P. megaterium* CIMT4 from maize rhizosphere soil in the Democratic Republic of Congo (2°20′08″S, 28°46′59″E; 1,734 meters above sea level). Soil tightly adhering to 1-month-old maize roots was collected, and 1.0 g was suspended in 9.0 mL of sterile 0.85% NaCl solution in water. A 100 µL aliquot was plated on tryptic soy agar (TSA) and incubated at 30°C for 24 hours. A single colony was streaked onto TSA three consecutive times to ensure purity, and a pure colony was subsequently cultivated in tryptic soy broth at 30°C for 24 hours with shaking at 200 rpm. Genomic DNA was extracted using the Wizard Genomic DNA Purification Kit (Promega, USA), and its quality and quantity were assessed with a Nanodrop One Spectrophotometer and Qubit 3.0 fluorometer (Thermo Fisher Scientific, USA).

The DNA was not sheared or size-selected prior to library preparation. The sequencing library was prepared using the SQK-LSK109 ligation kit (ONT, USA) and NEBNext companion module (New England Biolabs, USA) following the manufacturer’s guidelines. Long-read sequencing was conducted on the ONT MinION platform for 72 hours using a FLO-MIN111 (R10.3) flow cell at Kyungpook National University NGS Core Facility, Korea (10). FAST5 files were base-called with Guppy (v4.4.1) in high-accuracy mode, discarding reads with Phred scores < 7. The sequencing yielded 172,623 reads (N50: 7,181 bp). Adapter trimming was performed using Porechop (v0.2.4), *de novo* assembly was conducted with Flye (v2.9.5) (11), and the assembled contigs were annotated using NCBI’s Prokaryotic Genome Annotation Pipeline (PGAP v6.8) (12).

The genome of *P. megaterium* CIMT4 has a total length of 5,728,665 bp, assembled into eight contigs with an N50 value of 5,141,012 bp and an average coverage of 95×. It consists of a chromosome and seven plasmids. PGAP annotation identified 5,967 genes, including 5,705 protein-coding sequences (Table 1). Average nucleotide identity (ANI) analysis was conducted using the NCBI ANI pipeline (13) with default parameters to compare the genome against the reference genome of Priestia megaterium NBRC 15308 = ATCC 14581 (accession ID: GCA_006094495.1). The analysis showed 98.4% ANI and 85.26% assembly coverage, confirming the taxonomic classification as *Priestia megaterium*. CheckM v1.2.2 (14) with default parameters estimated 96.22% completeness and 2.35% contamination, indicating a high-quality genome assembly.

**TABLE 1:** Genome annotation features of *P. megaterium* CIMT4

Genome size	5.7 Mb
Number of contigs	8
Number of scaffolds	0
tRNA	123
rRNA	47
Plasmids	7
GC content	37.93
Contig L50	1
Contig N50	5.14 Mb
CDS	5,705

## Data Availability

The complete genome sequence data for *P. megaterium* CIMT4 strain and the plasmids have been deposited in the DDBJ/ENA/GenBank and can be accessed at https://www.ncbi.nlm.nih.gov/datasets/genome/GCF_042137975.1/ under the BioProject PRJNA1157567. The raw sequence data were deposited in the SRA database under the accession number SRR31373946.
